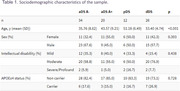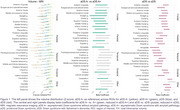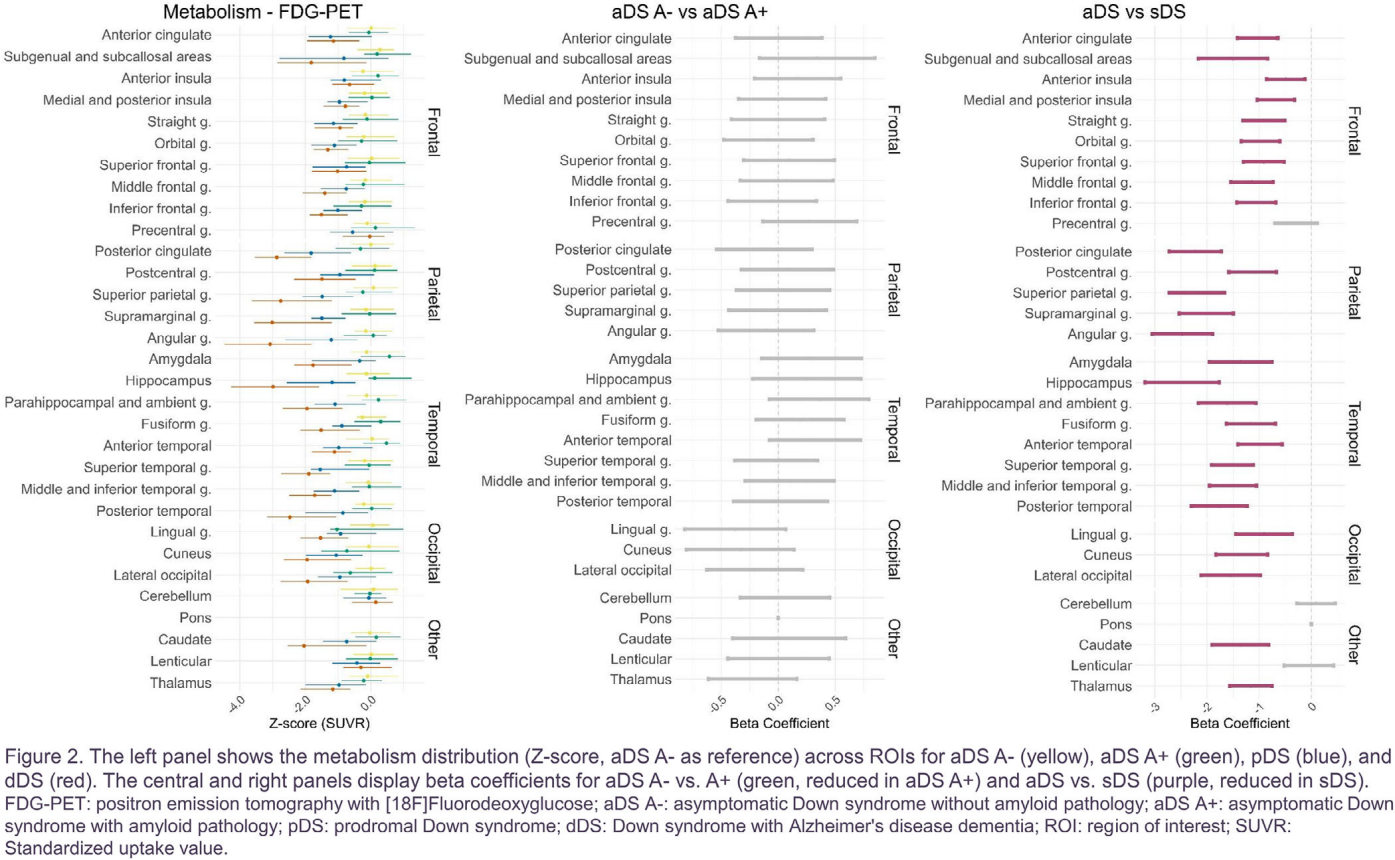# Distinct Sensitivity of MRI Versus [^18^F]FDG‐PET To Detect Cerebral Changes Across the Alzheimer's Continuum in Down Syndrome: A Multimodal Imaging Study

**DOI:** 10.1002/alz70856_107114

**Published:** 2026-01-11

**Authors:** José Enrique Arriola‐Infante, Alejandra O. Morcillo‐Nieto, Maria Franquesa‐Mullerat, Sara E Zsadanyi, Lídia Vaqué‐Alcázar, Mateus Rozalem Aranha, José Allende Parra, Zili Zhao, Javier Arranz, Íñigo Rodríguez‐Baz, Lucía Maure‐Blesa, Laura Videla, Isabel Barroeta, Laura Del Hoyo, Bessy Benejam, Susana Fernandez, Aida Sanjuan Hernandez, Sandra Giménez, Daniel Alcolea, Olivia Belbin, Albert Flotats, Valle Camacho, Alberto Lleó, Maria Carmona‐Iragui, Juan Fortea, Alexandre Bejanin

**Affiliations:** ^1^ Sant Pau Memory Unit, Hospital de la Santa Creu i Sant Pau, Institut de Recerca Sant Pau ‐ Universitat Autònoma de Barcelona, Barcelona, Spain; ^2^ Center of Biomedical Investigation Network for Neurodegenerative Diseases (CIBERNED), Madrid, Spain; ^3^ Department of Medicine, Faculty of Medicine and Health Sciences, Institute of Neurosciences, University of Barcelona, Barcelona, Spain. Institut d’Investigacions Biomèdiques August Pi i Sunyer (IDIBAPS), Barcelona, Spain; ^4^ Neuroradiology Section, Department of Radiology, Hospital de la Santa Creu i Sant Pau, Biomedical Research Institute Sant Pau, Universitat Autònoma de Barcelona, Spain, Barcelona, Spain; ^5^ Sant Pau Memory Unit, Department of Neurology, Hospital de la Santa Creu i Sant Pau, Institut d'Investigació Biomèdica Sant Pau (IIB SANT PAU), Facultad de Medicina ‐ Universitat Autònoma de Barcelona, Barcelona, Spain., Barcelona, Barcelona, Spain; ^6^ Sant Pau Memory Unit, Hospital de la Santa Creu i Sant Pau, Biomedical Research Institute Sant Pau, Universitat Autònoma de Barcelona, Barcelona, Spain; ^7^ Center for Biomedical Investigation Network for Neurodegenerative Diseases (CIBERNED), Madrid, Spain; ^8^ Barcelona Down Medical Center, Fundació Catalana Síndrome de Down, Barcelona, Spain; ^9^ Multidisciplinary Sleep unit. Hospital de la Santa Creu i Sant Pau, Institut d'Investigació Biomèdica Sant Pau (IIB SANT PAU), Barcelona, Spain; ^10^ Nuclear Medicine Department, Hospital de la Santa Creu i Sant Pau, Barcelona, Spain; ^11^ Nuclear Medicine Department, Hospital de la Santa Creu i Sant Pau, Barcelona, Barcelona, Spain

## Abstract

**Background:**

To date, limited evidence exists on the different sensitivity of MRI versus [^18^F]FDG‐PET to detect early cerebral changes along the Alzheimer's disease (AD) continuum in Down syndrome (DS). Therefore, we aimed to characterize volume and metabolism alterations in adults with DS and compare their performance in detecting AD clinical stages.

**Method:**

Cross‐sectional study, including 92 adults with DS from the Down‐Alzheimer Barcelona Neuroimaging Initiative (34 asymptomatic without amyloid pathology [aDS A−], 20 asymptomatic with amyloid pathology [aDS A+], 12 with prodromal AD [pDS], and 26 with AD dementia [dDS]; Table 1), who underwent 3T‐MRI and [^18^F]FDG‐PET. Amyloid status in the aDS group was determined using amyloid‐PET (>20 centiloids, *n* = 29) or CSF Aβ42/Aβ40 ratio (<0.062, Lumipulse, *n* = 25). Brain volume and metabolism values were extracted using the Hammers atlas, normalizing MRI volumes by total intracranial volume and PET images using the pons as reference region. We calculated standardized‐β coefficients, adjusted for sex and scanner, to assess the effectiveness of MRI and PET scans in identifying stages of AD (aDS A− vs. aDS A+; aDS vs. symptomatic DS [sDS=pDS + dDS]).

**Result:**

Progressive brain volume loss and metabolic decline were observed along the AD continuum in DS. Compared to aDS A−, aDS A+ exhibited lower volume in multiple regions, particularly in the frontal lobe. sDS individuals showed widespread atrophy, predominantly in temporal areas (Figure 1). In contrast, no significant hypometabolism was detected in aDS A+ compared to aDS A−, but sDS individuals exhibited global hypometabolism, primarily in temporo‐parietal regions (Figure 2).

**Conclusion:**

Amyloid pathology in aDS individuals is linked to frontal‐predominant atrophy without clear hypometabolism. In symptomatic stages, both MRI and [^18^F]FDG‐PET revealed widespread involvement, with atrophy predominating in temporal regions and hypometabolism in temporo‐parietal areas. Unlike in sporadic AD, no brain region showed greater hypometabolism than atrophy, suggesting alternative contributing factors in the general population. Our findings also suggest that MRI outperforms [^18^F]FDG‐PET in identifying brain changes associated with AD clinical stages in DS, which has implications for early diagnosis and clinical trials.